# Uncovering the ‘sphinx’ of sphingosine 1‐phosphate signalling: from cellular events to organ morphogenesis

**DOI:** 10.1111/brv.12798

**Published:** 2021-09-28

**Authors:** Mengqiao Cui, Verena Göbel, Hongjie Zhang

**Affiliations:** ^1^ Centre of Reproduction, Development and Aging, Faculty of Health Sciences University of Macau Taipa Macau SAR 999078 China; ^2^ Mucosal Immunology and Biology Research Center, Department of Pediatrics Massachusetts General Hospital and Harvard Medical School Boston MA 02114 U.S.A.; ^3^ MoE Frontiers Science Center for Precision Oncology University of Macau Taipa Macau SAR 999078 China

**Keywords:** sphingosine 1‐phosphate, signal transduction, extracellular mode, intracellular mode, membrane dynamics, organ morphogenesis

## Abstract

Sphingosine 1‐phosphate (S1P) is a bioactive sphingolipid metabolite, functioning as a signalling molecule in diverse cellular processes. Over the past few decades, studies of S1P signalling have revealed that the physiological activity of S1P largely depends on S1P metabolizing enzymes, transporters and receptors on the plasma membrane, as well as on the intracellular proteins that S1P binds directly to. In addition to its roles in cancer signalling, immunity and inflammation, a large body of evidence has identified a close link of S1P signalling with organ morphogenesis. Here we discuss the vital role of S1P signalling in orchestrating various cellular events during organ morphogenesis through analysing each component along the extracellular and intracellular S1P signalling axes. For each component, we review advances in our understanding of S1P signalling and function from the upstream regulators to the downstream effectors and from cellular behaviours to tissue organization, primarily in the context of morphogenetic mechanisms. S1P‐mediated vesicular trafficking is also discussed as a function independent of its signalling function. A picture emerges that reveals a multifaceted role of S1P‐dependent pathways in the development and maintenance of organ structure and function.

## I. INTRODUCTION

**Fig 1:**
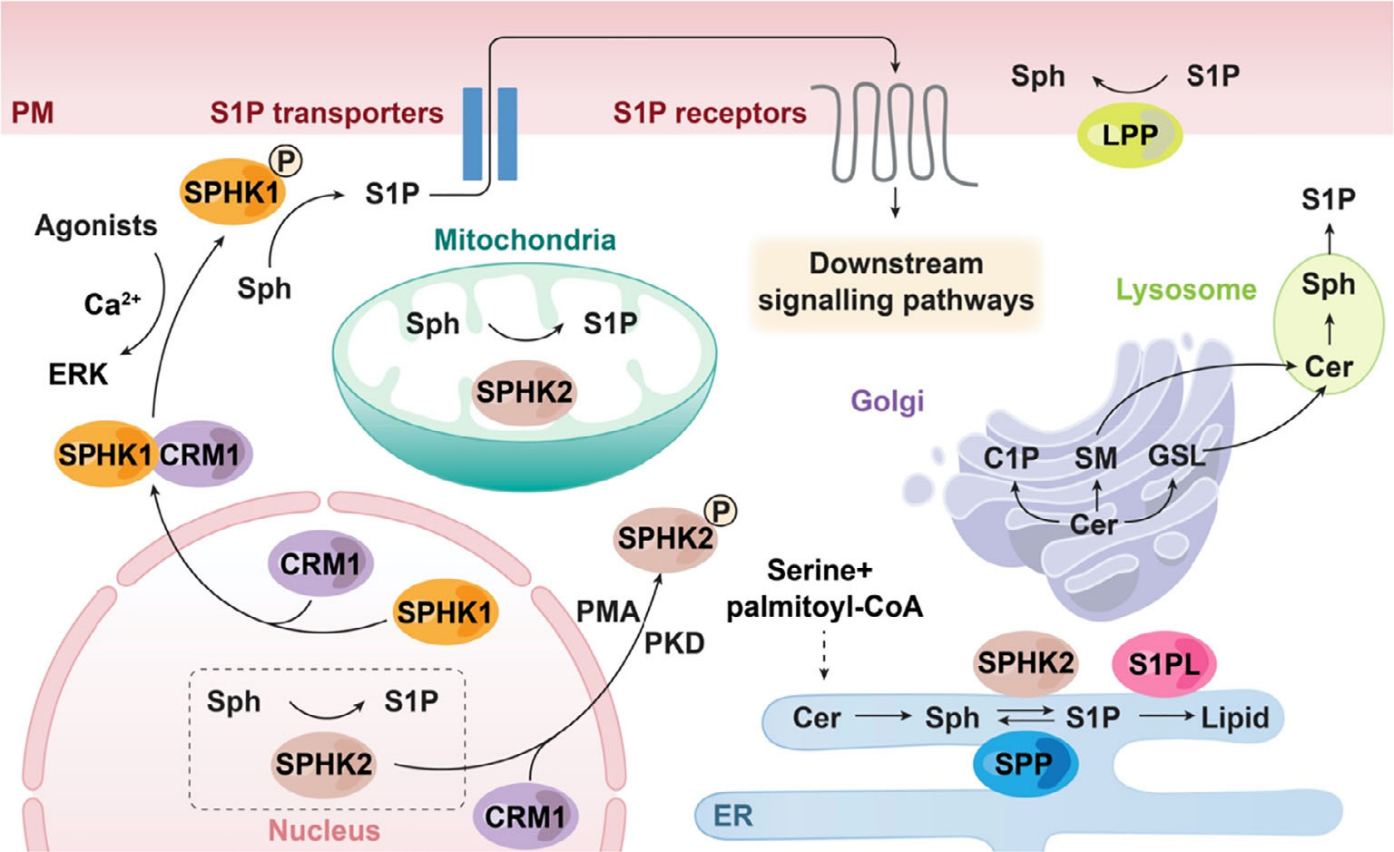
Overview of compartmentalized sphingosine 1‐phosphate (S1P) metabolism and inside–out signalling. S1P can be produced from *de novo* biosynthesis or from the salvage pathway of sphingolipid metabolism. In *de novo* sphingolipid biosynthesis, the condensation of serine and palmitoyl‐CoA at the endoplasmic reticulum (ER) generates ceramide (Cer), which can be converted to sphingosine (Sph) and then phosphorylated to S1P by sphingosine kinases (SPHKs). Ceramide can also be transported to Golgi to generate ceramide 1‐phosphate (C1P), sphingomyelin (SM) or glucosylceramide (GSL). In the salvage pathway, ceramide is generated through degradation of SM or GSL before converting to Sph and subsequently S1P, which occurs primarily in the lysosome. SPHK1 catalyses phosphorylation of Sph mainly at the cell plasma membrane (PM), and its translocation from the nucleus to the PM is driven by agonists through an extracellular signal‐related kinase (ERK) and Ca^2+^‐dependent SPHK1 phosphorylation. SPHK2 functions at the nucleus, ER and mitochondria, and its nuclear export depends on phorbol 12‐myristate 13‐acetate (PMA) and protein kinase D (PKD)‐mediated SPHK2 phosphorylation. The nuclear export of both SPHK1 and SPHK2 also depends on chromosomal region maintenance 1 (CRM1). Intracellular S1P is eventually degraded by either S1P lyase (SPL) or S1P phosphatases (SPPs). Extracellular S1P is dephosphorylated to sphingosine by lipid phosphate phosphatases (LPPs) localized at the PM. S1P induces a series of signalling pathways by activating S1P receptors after being exported outside cells by transporters, or by directly binding with intracellular protein targets.

Sphingolipids are essential lipid components making up the external leaflet of biological membranes in eukaryotic cells. While the majority of sphingolipids function as structural molecules by forming microdomains in the plasma membrane, some metabolites of sphingolipids such as sphingosine 1‐phosphate (S1P), act as a signalling molecule present at extremely low abundance (Hannun & Obeid, 2018). S1P can be derived from *de novo* biosynthesis or from the salvage pathway of sphingolipid metabolism. As depicted in Fig. 1, *de novo* sphingolipid biosynthesis starts from the condensation reaction of serine and palmitoyl CoA catalysed by serine palmitoyltransferases at the cytosolic leaflet of the endoplasmic reticulum (ER), leading to generation of ceramide (Futerman & Riezman, 2005; Hannun & Obeid, 2008; Breslow & Weissman, 2010). *De novo* synthesis also occurs in mitochondria with ceramide generated on both the outer and inner membranes of mitochondria (Bionda *et al*., 2004). Ceramide, the central hub of the sphingolipid network, can be deacylated into sphingosine and then phosphorylated by sphingosine kinases (SPHKs) to generate S1P or transported to the Golgi apparatus to generate ceramide 1‐phosphate, sphingomyelin or glucosylceramide and more complex glycosphingolipids (Futerman & Riezman, 2005). In the salvage pathway, ceramide is generated through degradation of sphingomyelin or glucosylceramide before converting to sphingosine, which occurs primarily in the lysosome and also on the plasma membrane (Hannun & Obeid, 2008). S1P is metabolized either through a dephosphorylation pathway by lipid phosphate phosphatases (LPPs) on the plasma membrane or S1P phosphatases (SPPs) at the ER to generate sphingosine, or through a degradation pathway by S1P lyase (S1PL) to generate long‐chain aldehyde and phosphoethanolamine.

S1P can exert signalling functions through extracellular and intracellular modes (Spiegel & Milstien, 2003; Takabe *et al*., 2008; Maceyka *et al*., 2012). In an extracellular mode, S1P is transported outside of cells *via* ATP‐binding cassette (ABC) transporters or major facilitator superfamily transporters, and selectively binds to G protein‐coupled receptors (GPCRs), which in turn triggers a cascade of downstream signalling pathways. Alternatively, S1P can act directly on some intracellular targets or as a membrane constituent to mediate coordinated cellular activities. Unlike growth factors and cytokines, lipid mediators are not encoded directly by the genome, thus the physiological activity of S1P is regulated by the spatiotemporal control of S1P biosynthesizing and metabolizing enzymes, transporters, receptors, and intracellular targets. S1P functions as a key signalling molecule in myriad cellular events, including cell proliferation, differentiation, adhesion, migration and death (Spiegel, Foster & Kolesnick, 1996; Spiegel & Milstien, 2003), all of which contribute to the development of various organs (Mendelson, Evans & Hla, 2014). Recently, new conceptual advances have underscored the plurality of the signalling events and effectors through which S1P acts during organ morphogenesis and delineated the exquisite specificity with which S1P targets selected receptors or intracellular modules for signalling. Herein, we provide an updated review on how S1P contributes to various cellular behaviours as well as their subsequent assembly into tissues, with a focus on the latest insights into the impact of intersecting signal transduction pathways underlying the coordinated cellular events for organ development. We discuss findings associated with the role of S1P in organ morphogenesis through its extracellular and intracellular actions as well as through membrane trafficking. For specific aspects of the importance of S1P in physiological processes or pathogenesis and therapeutic intervention of various disease conditions, including cancer, inflammation and fibrosis, the reader is referred to several excellent reviews (Pyne & Pyne, 2010; Spiegel & Milstien, 2011; Maceyka *et al*., 2012; Kunkel *et al*., 2013; Maceyka & Spiegel, 2014; Proia & Hla, 2015; Pyne, Adams & Pyne, 2016; Huwiler & Zangemeister‐Wittke, 2018; Ogretmen, 2018; Cartier & Hla, 2019).

## II. ROLE OF S1P METABOLISM IN ORGAN MORPHOGENESIS

**Fig 2:**
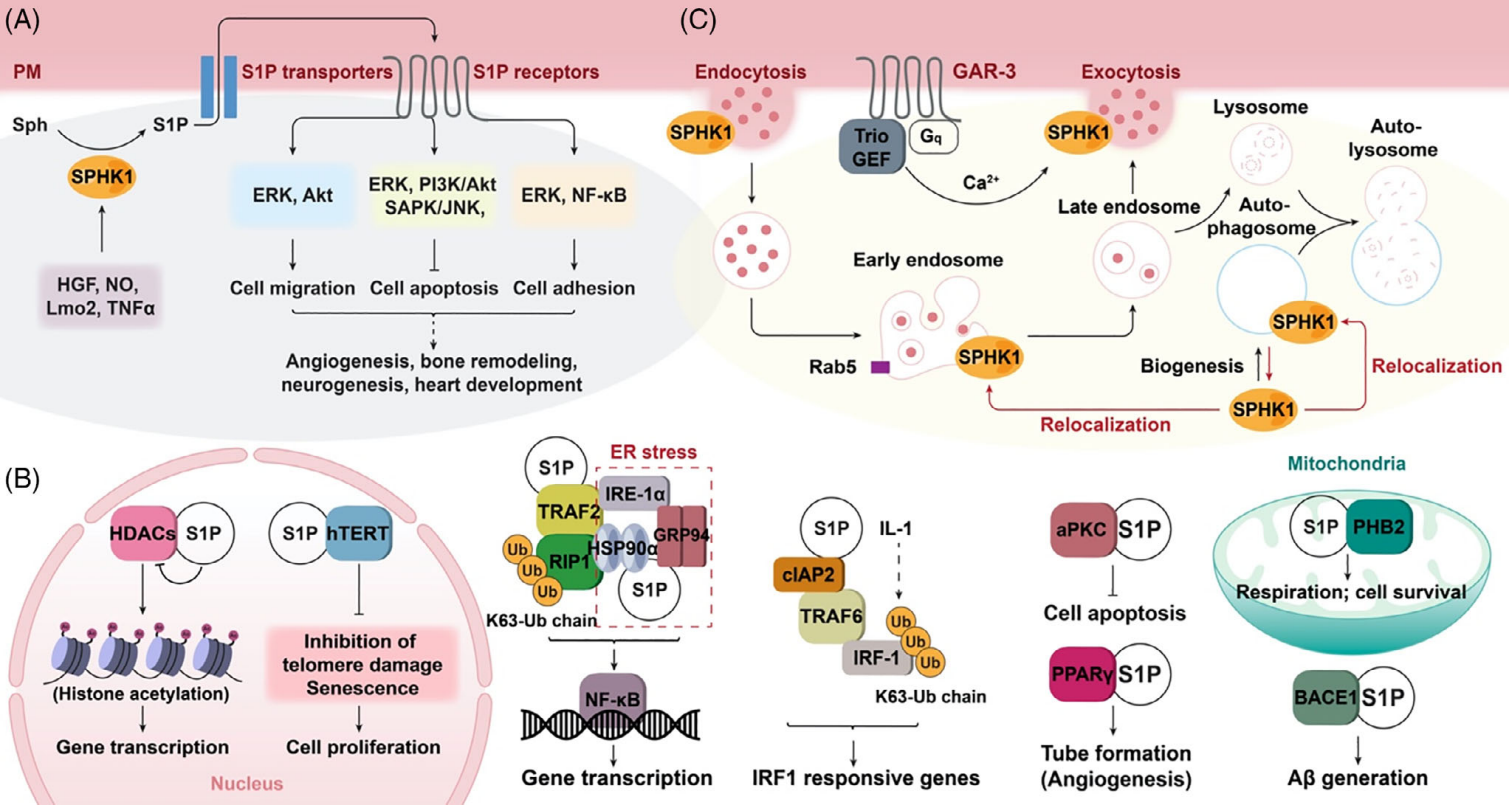
Role of sphingosine kinase (SPHK)‐mediated sphingosine 1‐phosphate (S1P) signalling in organ morphogenesis. (A) Extracellular mode: activation of SPHK1 by hepatocyte growth factor (HGF), nitric oxide (NO), LIM‐domain only 2 (Lmo2) and tumor necrosis factor α (TNFα) promotes S1P generation and subsequent export *via* transporters. Extracelluar S1P elicits signals through S1P receptors (S1PRs) to stimulate cell migration, stabilize cell adhesion and inhibit cell apoptosis. These cellular events are involved in organ morphogenesis including angiogenesis, bone morphogenesis, neurogenesis and heart development. Akt, protein kinase B; ERK, extracellular signal‐regulated kinase; JNK, c‐Jun N‐terminal kinase; NF‐κB, nuclear factor kappa light‐chain‐enhancer of activated B cells; PI3K, phosphoinositide 3‐kinase; SAPK, stress‐activated protein kinase. (B) Intracellular mode: S1P binds to intracellular targets in different cellular compartments. In the cytoplasm, SPHK1‐generated S1P directly binds to the E3 ligases TNF receptor‐associated factor 2 (TRAF2) and cIAP2, promotes receptor interacting protein 1 (RIP1) and interferon regulatory factor 1 (IRF1) polyubiquitination, and activates the NF‐κB pathway and IRF1‐responsive genes, respectively. Upon endoplasmic reticulum (ER) stress the S1P–TRAF2–RIP1 complex can associate with the stress‐responsive proteins, heat shock protein 90α (HSP90α), heat shock protein 90β family member 1 (GRP94) and ER to nucleus signalling 1α (IRE1α). S1P also binds with atypical protein kinase C (aPKC), β‐site amyloid precursor protein cleaving enzyme‐1 (BACE1) and peroxisome proliferator‐activated receptor γ (PPARγ) to protect the cell from apoptosis, to stimulate amyloid‐β peptide (Aβ) production (the main cause of Alzheimer's disease) and to promote tube formation in angiogenesis, respectively. In the nucleus, SPHK2‐generated S1P forms a co‐repressor complex with histone deacetylases (HDACs) and reduces HDAC activity. Reduced HDAC activity increases histone acetylation and regulates gene expression. S1P binding to nuclear human telomerase reverse transcriptase (hTERT) contributes to inhibition of telomere damage and senescence, thus promoting cell proliferation. In mitochondria, SPHK2‐generated S1P binds to prohibitin 2 (PHB2) to regulate respiration, cardioprotection, ER stress and apoptosis. IL‐1, interleukin 1; Ub, ubiquitin. (C) Vesicular trafficking: SPHK1 regulates membrane trafficking. Upon activation, SPHK1 targets to endocytic tubular invagination and Ras‐related protein (Rab5)‐positive endosomes. SPHK1/S1P‐mediated endocytic trafficking also converges with autophagy. SPHK1 enhances autophagy biogenesis and function, and autophagy, in turn, stimulates SPHK1 re‐localization to the endosome/autophagosome. SPHK1 is also localized to the membrane in a muscarinic acetylcholine receptor (GAR‐3)‐induced calcium signalling‐dependent manner for release of acetylcholine. G_q_, heterotrimeric G protein alpha subunit; TrioGEF, triple functional domain protein guanine nucleotide exchange factor.

SPHK‐generated S1P can function as a ligand of GPCRs, as a second messenger and in directing membrane dynamics (Fig. 2). In this section and Section III, we explore the molecular, cellular and tissue‐level events that define S1P as a paracrine factor in organ morphogenesis primarily through regulating its biosynthesis, catabolism and transport (Fig. 2A; Table 1). The function of S1P as a second messenger (Fig. 2B) and the roles of SPHK/S1P in membrane trafficking (Fig. 2C) will be discussed in Sections IV and V, respectively.

**Table 1:** Roles of sphingosine 1‐phosphate (S1P) catabolism in organ morphogenesis

Enzyme	Organism	Organs affected	Cellular events mediated	Signaling involved	References
SPP1	Mouse	Skin	Regulates keratinocyte differentiation and epidermal homeostasis	Ca^2+^ signalling	Allende *et al*. (2013)
SPP2	Mouse	Pancreas	Regulates islet β‐cell differentiation and protects cells from ER stress	—	Taguchi *et al*. (2016)
	Mouse and human	Intestine	Promotes disruption of mucosal barrier function	—	Huang *et al*. (2016)
S1PL	*Drosophila melanogaster*	Muscle and reproductive system	Regulate cell apoptosis	*reaper*, *hid* and *grim*	Herr *et al*. (2003) Phan *et al*. (2007)
	*Caenorhabditis elegans*	Reproductive system and intestine	—	—	Mendel *et al*. (2003)
	Mouse	Lung, heart, bone, thymus and nervous system	Bone: suppresses osteoclastogenesis and stimulates osteoblastogenesis Nervous system: regulates cell apoptosis	Bone: calcitonin; p38–GSK3β–β‐catenin; WNT5A–LRP5 Nervous system: Ca^2+^ signalling; calpain, caspases; CDK5	Vogel *et al*. (2009); Keller *et al*. (2014); Weske *et al*. (2018, 2019); Hagen *et al*. (2011)
LPP1	Mouse	Hair and reproductive system	—	—	Yue *et al*. (2004)
LPP3	Mouse	Angiogenesis, vasculogenesis and axis patterning	Regulates vasculogenesis and axis patterning	WNT signalling; β‐catenin‐mediated TCF transcription	Escalante‐Alcalde *et al*. (2003)
	*Xenopus laevis*	Axis patterning	—	WNT signalling	

CDK5, cyclin‐dependent kinase 5; ER, endoplasmic reticulum; GSK3β, glycogen synthase kinase 3β; LPP, lipid phosphate phosphatase; LRP5, lipoprotein receptor‐related protein; p38, mitogen‐activated protein kinase 14, also known as MAPK14; SPP, S1P phosphatase; S1PL, S1P lyase; TCF, transcription factor; WNT, wingless‐related integration site.

### (1) S1P biosynthetic enzymes – sphingosine kinases (SPHKs)

S1P is biosynthesized by SPHKs through the phosphorylation of sphingosine, and SPHKs are conserved across diverse organisms (Spiegel & Milstien, 2007). There are two isoforms, SPHK1 and SPHK2, in mammals, each displaying distinct yet overlapping functions in a subcellular compartment‐dependent manner (Fig. 1). SPHK1 is predominantly localized in the cytoplasm and SPHK2 in the nucleus, although they can both shuttle between the nucleus and cytoplasm in a *cis*‐regulatory element [the nuclear export signal (NES)] sequence‐ and *trans*‐acting factor [nuclear export receptor chromosomal region maintenance 1 (CRM1)]‐dependent manner (Inagaki *et al*., 2003; Ding *et al*., 2007). Interestingly, SPHK1 can translocate to the plasma membrane driven by numerous agonists [such as cytokines, growth factors and phorbol 12‐myristate 13‐acetate (PMA)] and proteins (such as calcium and integrin‐binding protein 1 and filamin A) through inducing extracellular signal‐regulated kinase (ERK)‐dependent SPHK1 phosphorylation in a Ca^2+^/camodulin dependent manner (Pitson *et al*., 2003; Maceyka *et al*., 2008; Jarman *et al*., 2010) (Fig. 1). The export of SPHK2 to cytoplasm depends on PMA‐induced, protein kinase D‐mediated SPHK2 phosphorylation (Ding *et al*., 2007). SPHK2 is also present in mitochondria, ER and other intracellular phosphoinositide‐associated compartments (Don & Rosen, 2009; Strub *et al*., 2011), suggesting an even greater diversity of functions.

**Fig 3:**
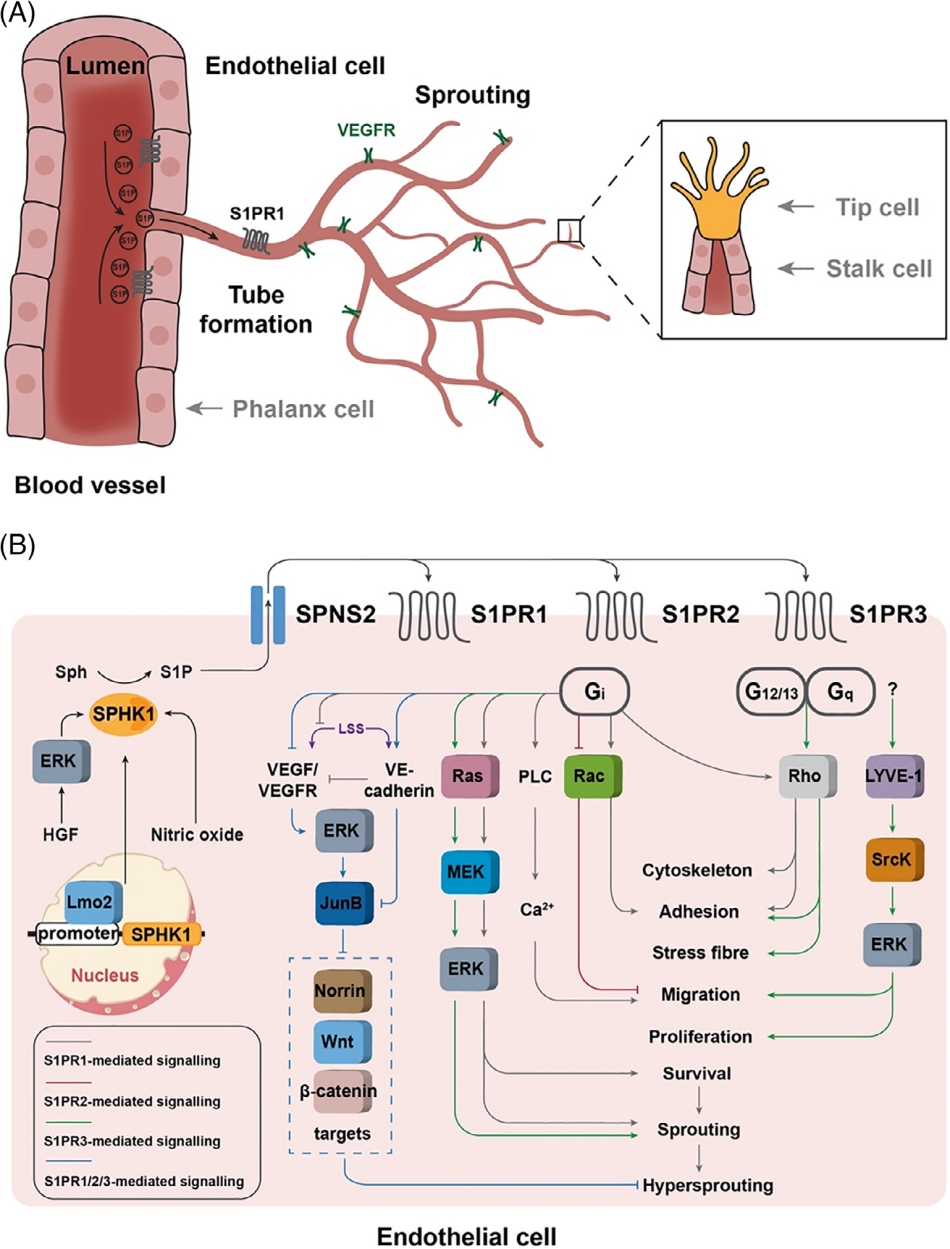
Sphingosine 1‐phosphate (S1P) signalling in angiogenesis. (A) Angiogenesis is the growth of new blood vessels through sprouting, branching and pruning of pre‐existing blood vessels. Angiogenesis is regulated by balanced signalling between S1P and the angiogenic growth factor vascular endothelial growth factor (VEGF) through S1P receptors (S1PRs) and VEGF receptors (VEGFRs), respectively. In response to the combined regulation of the signalling depicted here as well as many other factors (not shown due to a lack of evidence for a direct link with S1P), the tip cells migrate to direct sprouting, the stalk cells proliferate to enable lumenogenesis and the phalanx cells remain quiescent to stabilize the single‐cell epithelial layer during angiogenesis. Note that S1PR1 is activated not only by blood‐derived S1P but also by biomechanical signals [laminar shear stress (LSS)]. (B) S1P is generated in endothelial cells by sphingosine kinase 1 (SPHK1), whose activity is subject to regulation by various factors, including hepatocyte growth factor (HGF) through extracellular signal‐regulated kinase (ERK1/2)‐mediated SPHK1 phosphorylation, nitric oxide and transcription factor LIM‐domain only 2 (Lmo2). SPHK1‐generated S1P is exported by S1P transporter spinster homolog 2 (SPNS2) and functions as a paracrine factor to trigger signalling pathways for angiogenesis through G protein‐coupled S1PRs. S1PR1 stabilizes cell adhesion and cytoskeleton through rat sarcoma (Ras)‐related C3 botulinum toxin substrate (Rac) and Ras homolog family member (Rho), promotes cell migration [in a phospholipase C (PLC)/Ca^2+^‐dependent manner], and endothelial cell survival and vessel sprouting (*via* activating Ras/ERK). S1PR1 also functions to suppress hypersprouting by (1) inhibiting LSS‐dependent VEGF/VEGFR signalling; (2) generating a spatial gradient of Jun B proto‐oncogene (JunB) together with S1PR2 and S1PR3 for endothelial cell specialization. The JunB gradient is generated through inducing JunB expression at the sprouting vascular region *via* VEGF/VEGFR2, and suppressing JunB expression in the nascent vascular network *via* vascular endothelial (VE)‐cadherin, which, in turn, down‐regulates a subset of Norrin/wingless‐related integration site (Wnt)/β‐catenin‐regulated genes. In contrast to S1PR1, S1PR2 inhibits cell migration by downregulating Rac. S1PR3 stabilizes cell adhesion and stress fibre formation *via* Rho, and stimulates cell migration and proliferation [through lymphatic vessel endothelial HA receptor (LYVE‐1)/Src proto‐oncogene kinase (SrcK)/ERK signalling] as well as vessel sprouting (*via* mitogen‐activated protein kinase kinase (MEK)/ERK signalling). G_i_, G_12/13_ and G_q_, heterotrimeric G protein alpha subunits.

SPHKs exert a series of cellular functions required for organ morphogenesis through generating S1P (Fig. 2A). First, SPHKs regulate cell migration in diverse cell types. For example, SPHK1 promotes cell motility in human lung microvascular endothelial cells (HLMVECs) through hepatocyte growth factor (HGF)‐induced ERK1/2‐mediated SPHK‐1 phosphorylation and accumulation at the cell periphery. SPHK1 co‐localizes with actin and cortactin in lamellipodia at the cell periphery where locally generated S1P is transported extracellularly *via* S1P transporter spinster homolog 2 (SPNS2) and signals as a paracrine factor through S1P receptor 1 (S1PR1) to direct cell migration (Laurenzana *et al*., 2015; Fu *et al*., 2016). SPHK1 can be activated by nitric oxide (NO) leading to S1P upregulation, and subsequently induces migration of human umbilical vein endothelial cells (HUVECs) and triggers tube formation for angiogenesis *via* S1PR3 (Schwalm, Pfeilschifter & Huwiler, 2010). SPHK1‐mediated endothelial cell (EC) migration is also subject to regulation by the transcription factor LIM‐domain only 2 (Lmo2) which binds directly to the SPHK1 promoter and regulates SPHK1 expression as well as EC migration and vascular development in the HUVEC cell line, and mouse and zebrafish (*Danio rerio*) models (Fig. 3) (Matrone *et al*., 2017). Moreover, SPHK1/S1P‐induced protein kinase B (Akt/Pkb) phosphorylation enhances hepatic stellate cell (HSC) migration and fibrogenesis in a fibronectin–integrin‐ and dynamin‐dependent manner (Wang *et al*., 2015, 2017).

Both SPHK1 and SPHK2 also promote migration and capillary morphogenesis of human mesenchymal progenitor mesoangioblasts (Laurenzana *et al*., 2015). While mounting evidence has pinpointed a positive role of SPHK/S1P in positively regulating cell motility, SPHK2 was reported to suppress cell migration in renal mesangial cells and fibroblasts (from SPHK2‐knockout mice) through ERK and phosphoinositide 3‐kinase (PI3K)/Akt cascades and the small G protein rat sarcoma (Ras) homolog family member A (RhoA) (Schwalm *et al*., 2015). These seemingly conflicting results are probably attributable to a compensatory upregulation of SPHK1 and S1PR3 in SPHK2 knockout cells, eventually leading to a net increase in S1P signalling (Schwalm *et al*., 2015).

SPHK/S1P also functions to protect cells from apoptosis. SPHK/S1P was first discovered to suppress ceramide‐mediated programmed cell death through stimulating the ERK pathway, counteracting the ceramide‐induced activation of stress‐activated protein kinase (SAPK/JNK) in human promyelocytic HL‐60 cells, U937 monoblastic leukemia cells and Swiss 3T3 cells (Cuvillier *et al*., 1996). A suppression effect of SPHK1 on ceramide‐mediated apoptosis was also confirmed in NIH 3T3 fibroblasts, HEK 293 cells and Jurkat T cells (Olivera *et al*., 1999). In HUVECs, SPHK1 overexpression activates the PI3K/Akt pathway, upregulates the anti‐apoptotic protein B‐cell lymphoma 2 (BCL2) and down‐regulates the proapoptotic protein BCL2 interacting mediator of cell death (BIM) in a platelet EC adhesion molecule‐1 (PECAM1)‐dependent manner, thereby leading to enhanced EC survival (Limaye *et al*., 2005). Consistently, overexpression of SPHK2 activates autophagy through directly interacting with BCL2 *via* its BH3 domain and subsequently dissociating it from Beclin‐1, and thereby increases murine neural cell viability upon ischemic injury (Song *et al*., 2017). Similar to the opposing effects of SPHK1 and SPHK2 on migration, other studies have suggested an opposite function for SPHK2 in inducing apoptosis and suppressing proliferation (Liu *et al*., 2003; Okada *et al*., 2005). For example, in renal mesangial cells derived from SPHK2 transgenic mouse, SPHK2 overexpression suppresses proliferation and promotes stress‐induced apoptosis, probably through reducing ERK and Akt activation and antiapoptotic B‐cell lymphoma‐extra large (BCL‐XL) expression and increasing caspase‐9 activity (Beyer *et al*., 2018), whereas SPHK2 knockout enhances proliferation and protects the renal mesangial cells from stress‐induced apoptosis (Schwalm *et al*., 2015). Although this discrepancy may be partially ascribable to compensatory effects of two SPHKs as discussed above for migration, diverse other factors, including distinct subcellular localizations of the two SPHK isoforms, the intracellular or extracellular action mode of S1P, as well as cell type, cell state and culture conditions may also be contributing factors, with a detailed explanation awaiting further exploration.

SPHKs are a potent regulator for cell adhesion. Stimulation of activity of SPHKs in ECs by tumor necrosis factor α (TNFα) enhances ERK and nuclear factor kappa‐light‐chain‐enhancer of activated B cells (NF‐kB) signalling and increases vascular adhesion molecule‐1 (VCAM‐1) and E‐selectin expression (Xia *et al*., 1998), whereas inhibition of SPHK1 reduces adiponectin‐induced activation of NF‐kB signalling and thereby decreases the expression of adhesion proteins including VCAM‐1, intracellular adhesion molecule‐1 (ICAM‐1), E‐selectin and monocyte chemoattractant protein‐1 (MCP‐1) in vascular ECs (Kase *et al*., 2007).

Blood vessel development involves the *de novo* formation of a primitive vascular network from the differentiated ECs (vasculogenesis) followed by vascular sprouting and remodelling into a complex network (angiogenesis) (Masuda & Asahara, 2003; Rafii & Lyden, 2003). Specifically, angiogenesis relies on orchestrated activity of migration of tip cells leading the vascular forefront and proliferation of stalk cells elongating the sprout as well as survival of phalanx cells lining quiescent vessels (Fig. 3A) (Vandekeere, Dewerchin & Carmeliet, 2015). Thus, S1P‐mediated EC migration, proliferation, survial and adhesion contribute directly to blood vessel development. Consistently, although single knockout mice are superficially normal due to redundant function of the two SPHKs, double SPHK1‐ and SPHK2‐knockout mice cannot survive past the embryonic stage with severely disrupted neurogenesis and angiogenesis, at least in part due to increased cell death and decreased cell proliferation (Mizugishi *et al*., 2005; Michaud *et al*., 2006; Xiong *et al*., 2014). Red blood cell (RBC)‐specific depletion of SPHK1 and SPHK2 in mice also leads to early embryonic lethality accompanied by vascular and cardiac defects, which can be partially rescued upon S1PR1 activation (Xiong *et al*., 2014), highlighting an important role of erythrocyte‐derived S1P in vascular development.

S1P is strictly required in cardiac development. Reducing S1P levels through dimethylsphingosine treatment led to loss of heart cellularity and cardiac cushions in whole‐mouse embryo culture, and increasing S1P levels through exogenous supplement inhibited cell migration and blocked endothelial to mesenchymal transformation in atrioventricular canal culture (Wendler & Rivkees, 2006). As further evidence for the role of S1P in heart development, a maternal‐zygotic *sphk2* mutant, but not a maternal‐zygotic *sphk1* mutant or maternal *sphk2* mutant, display a defect in cardiac progenitor migration and thereby cardia bifida in zebrafish (Hisano *et al*., 2015), a similar phenotype to that observed in mutants of the S1P transporter SPNS2 and S1P receptor S1PR2 (Kupperman *et al*., 2000; Kawahara *et al*., 2009).

**Fig 4:**
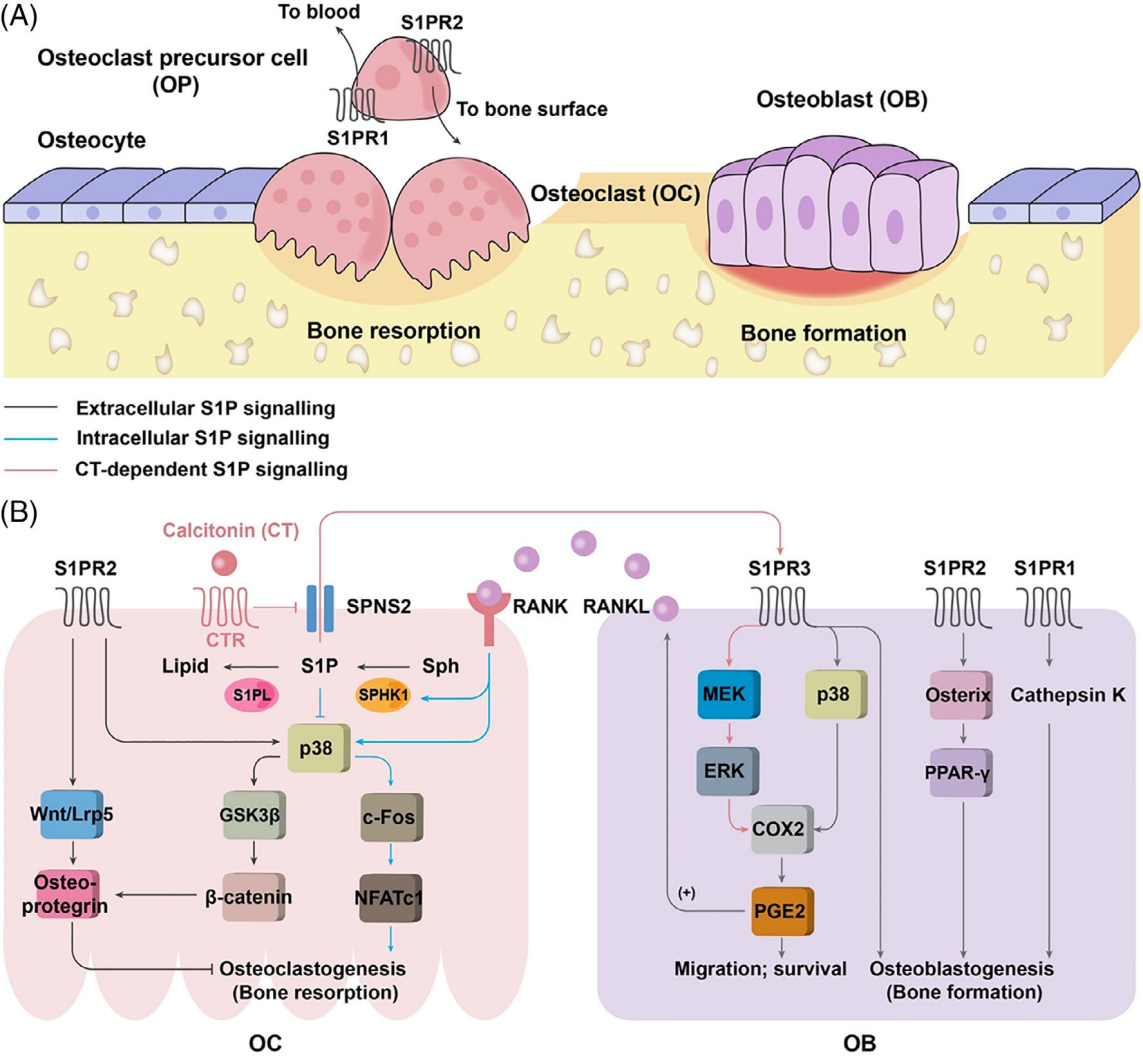
Sphingosine 1‐phosphate (S1P) signalling in bone morphogenesis. (A) Bone morphogenesis involves a highly dynamic regulation of bone resorption and formation by osteoclasts (OCs) and osteoblasts (OBs). Extracellular S1P secreted from osteoclast precursor cells promotes circulation of OCs between blood and bone through S1P receptor 1 (S1PR1)‐directed OCs migrating away from bone and S1PR2‐mediated OCs migrating towards bone. (B) Osteoclastogenesis is inhibited by both extracellular S1P signalling [through S1PR2/wingless‐related integration site (Wnt)/lipoprotein receptor‐related protein 5 (Lrp5) and S1PR2/glycogen synthase kinase 3β (GSK3β)/β‐catenin‐induced osteoprotegrin upregulation] and intracellular S1P signalling [through negative regulation of the p38/FBJ osteosarcoma oncogene (c‐Fos)/nuclear factor of activated T cells 1 (NFATc1) pathway]. Extracellular S1P secreted from OCs [negatively regulated by calcitonin (CT) and calcitonin receptor (CTR)] promotes the migration and survival of OBs by cyclooxygenase‐2 (COX2) and prostaglandin E2 (PGE2)‐induced receptor activator of NF‐κB ligand (RANKL) upregulation through modulating activities of p38 and extracellular signal‐regulated kinase (ERK) in OBs. RANKL secreted from OBs binds with RANK in OCs and activates SPHK1, which, together with S1P lyase (S1PL), in turn regulates S1P metabolism. In addition, S1PR1‐ and S1PR2‐mediated S1P signalling stimulates osteoblastogenesis by regulating osterix/peroxisome proliferator‐activated receptor γ (PPARγ) and cathepsin K, respectively. MEK, mitogen‐activated protein kinase kinase; SPNS2, S1P transporter spinster homolog.

A study using bone marrow‐derived macrophage (BMM) single‐ and BMM/osteoblast co‐culture systems revealed a vital role of SPHK1/S1P signalling in bone development. Throughout bone development, maintaining balanced bone remodelling between osteoclast‐mediated bone resorbing and osteoblast‐dependent bone formation is essential (Fig. 4A). SPHK‐S1P plays a dichotomous role in this process. Extracellular S1P secreted from osteoclast precursor cells promotes osteoblastogenesis as well as migration and survival of osteoblasts by cyclooxygenase‐2 (COX2) and prostaglandin E2 (PGE2)‐induced receptor activator of NF‐kB ligand (RANKL) upregulation through modulation of p38 and ERK activities in osteoblasts, whereas intracelluar S1P produced upon RANKL‐mediated SPHK1 activation negatively regulates the osteoclast differentiation program in the absence of osteoblasts by inhibiting the downstream signals p38/FBJ osteosarcoma oncogene (c‐Fos)/nuclear factor of activated T cells 1 (NFATc1) (Fig. 4B) (Ryu *et al*., 2006).

Taken together, through shuttling among the nucleus, cytoplasm and cell membranes, SPHK1 and SPHK2 function to promote cell migration, proliferation, differentiation and adhesion and to suppress apoptosis, which in turn, contribute to the development of diverse organs/tissues including neurogenesis and angiogenesis, bone remodelling and cardiac development (Fig. 2A).

### (2) S1P catabolic enzymes

S1P can be metabolized by three types of enzymes: SPPs, S1PL and LPPs. SPPs and S1PL are located at the ER with their active sites facing the luminal and cytosolic sides, respectively; thus they function to degrade intracellular S1P (Kihara *et al*., 2003; Ikeda, Kihara & Igarashi, 2004). LPP enzymes are located on the plasma membrane with the active site facing outside the cell and are mainly responsible for degradation of S1P in extracellular media (Kai *et al*., 2006) (Fig. 1).

#### (a) S1P phosphatases (SPPs)

SPPs are responsible for turning S1P into sphingosine by dephosphorylation. Mammalian SPP has two isoforms, SPP1 and SPP2 (encoded by genes *Sgpp1* and *Sgpp2*, respectively). Mouse SPP1 is expressed in various tissues, and SPP2 is mainly expressed in the stomach, colon and intestine (Johnson *et al*., 2003; Huang *et al*., 2016). Knockdown of *Sgpp1* leads to intracellular accumulation of S1P and increased secretion of S1P into media, and consequently, increased resistance to cytotoxic agents such as TNFα and daunorubicin (Johnson *et al*., 2003). By contrast, SPP1‐overexpressing cells upon S1P supplementation display accumulation of ceramide in ER and resulting defects in transporting proteins from ER to Golgi (Giussani *et al*., 2006). *In vivo* studies have revealed that SPP1 and SPP2 have distinct fucntions. *Sgpp1*‐knockout mice exhibit neonatal lethality and abnormal skin characterized by desquamation and epidermal hyperplasia. Keratinocytes derived from the skin of *Sgpp1* ^−/−^ mice show increased intracellular levels of S1P and Ca^2+^, as well as upregulation of genes involved in keratinocyte differentiation, demonstrating an essential role of SPP1 in keratinocyte differentiation and epidermal homeostasis through mediating Ca^2+^ signalling (Allende *et al*., 2013). In contrast to *Sgpp1* ^−/−^ mice, *Sgpp2* ^−/−^ mice are viable into adulthood and have normal skin. Instead they exhibit defects in adaptive proliferation and elevated ER stress in pancreatic islet β‐cells, revealing SPP2 as an important factor linking S1P‐mediated ER stress with dysfunction of β‐cells and pathogenesis of diabetes (Taguchi *et al*., 2016). In mice and humans, SPP2, but not SPP1, promotes mucosal integrity disruption and contributes to the pathogenesis of colitis in the gastrointestinal tract. Down‐regulation of SPP2 attenuated lipopolysaccharide (LPS)‐induced paracellular permeability in cultured cells and enhanced expression of the adherens junction protein E‐cadherin (Huang *et al*., 2016).

#### (b) S1P lyase (S1PL)

S1PL catalyses the cleavage of S1P at the C2–C3 carbon–carbon bond to generate phosphoethanolamine and hexadecenal, both of which are precursors of glycerophospholipids (Le Stunff *et al*., 2002). Conceivably, S1PL activity affects not only S1P metabolism, but also the conversion of sphingolipid to glycerophospholipid. S1PL is expressed highly in the small intestine, thymus and spleen, moderately in the liver, kidney, lung, stomach and testis, and barely in the brain, heart and skeletal muscle of the mouse and rat (Ikeda *et al*., 2004). S1PL is essential during development in various animals. In *Drosophila melanogaster*, null mutation (*sply* ^*05091*^) of *sply* (encoding *Drosophila* S1PL) causes a wide range of developmental defects including larval lethality, abnormal dorsal longitudinal flight muscles, altered reproductive structures and elevated apoptosis in both embryo and gonad (Herr *et al*., 2003; Phan *et al*., 2007). These phenotypes are suppressed by either reducing sphingolipid intermediates *via* a second‐site mutation in the serine palmitoyltransferase subunit lace/SPTLC2 (*lace* ^*k05305*^) or by genetic disruption of the proapoptotic genes *reaper*, *hid and grim*, revealing the convergence of cell apoptosis and sphingolipid metabolism in S1PL‐mediated organ development and growth (Phan *et al*., 2007). In *Caenorhabditis elegans*, inhibition of S1PL (encoded by *spl‐1*) caused poor feeding, delayed growth, and reproductive and intestinal defects due to accumulation of phosphorylated and unphosphorylated long‐chain bases (Mendel *et al*., 2003). *Sgpl1*‐deficient mice exhibited developmental abnormalities and lethality during the perinatal period (Vogel *et al*., 2009), which can be partially rescued by expression of human S1PL. Moreover, global *Sgpl1* deficiency leads to a high‐bone‐turnover phenotype with increased osteoclastogenesis and bone mass in newborn mice (Keller *et al*., 2014). Interestingly, deletion or inhibition of S1PL in adult mice suppresses osteoclastogenesis and stimulates osteobalstogenesis, thereby phenocopying the high‐bone‐mass phenotype observed in newborn global *Sgpl1*‐knockout mice, as well as successfully rescuing severe genetic osteoporosis caused by osteoprotegerin deficiency (Weske *et al*., 2018, 2019). These data demonstrated a role of S1P signalling in osteoanabolic and antiresorptive processes, consistent with a recognized positive role of S1P in bone formation through *in vitro* study of SPHKs. In the nervous system of *Sgpl1*‐deficient mice, S1P induces neuronal death through triggering intracellular calcium signalling, which, in turn, induces a calpain‐mediated ER stress‐specific caspase cascade and activates the cyclin‐dependent kinase CDK5 (Hagen *et al*., 2011).

#### (c) Lipid phosphate phosphatases (LPPs)

LPPs are integral membrane enzymes, and similar to SPPs, also function to metabolize S1P through dephosphorylation, although they have a wide range of substrates including phosphatidate, lysophosphatidate, ceramide 1‐phosphate and diacylglycerol pyrophosphate in addition to S1P. Mammals have three isoforms of LPPs, LPP1, LPP2 and LPP3 (encoded by the genes *Ppap2a*, *Ppap2c* and *Ppap2b*, respectively). LPP1 and LPP3 are ubiquitously expressed in all tissues throughout development, and functional analyses have revealed essential roles of LPP3 in vasculogenesis and embryonic anterior–posterior axis patterning (Zhang, Sundberg & Gridley, 2000; Escalante‐Alcalde *et al*., 2003; Yue *et al*., 2004; Panchatcharam *et al*., 2013, 2014). However, the links between organ defects and altered S1P signalling await further investigation, given the wide range of LPP substrates.

In summary, S1P catabolic enzymes with spatially separated localization remove both intra‐ and extracellular S1P. S1P signalling mediated specifically through S1P catabolic enzymes is required for development of the pancreas, bone, neurons, and reproductive and vascular systems (Table 1).

## III. ROLE OF EXTRACELLULAR S1P SIGNALLING IN ORGAN MORPHOGENESIS

In extracellular S1P signalling, S1P must be exported extracellularly by transporters, and S1P elicits signals through the transmembrane protein GPCRs (Spiegel & Milstien, 2003).

### (1) S1P transporters

Multiple types of transporters, including ABC transporters and major facilitator superfamily transporters, participate in S1P export.

#### (a) ATP‐binding cassette (ABC) transporters

ABC transporters play an important role in transporting lipids across the cell membrane, among which subfamily members ABCA1, ABCB1, ABCC1 and ABCG2 contribute to S1P export (Honig *et al*., 2003; Mitra *et al*., 2006; Sato *et al*., 2007; Gratschev *et al*., 2009; Kobayashi *et al*., 2009; Takabe *et al*., 2010; Japtok *et al*., 2012; Cartwright *et al*., 2013). ABCC1 has been widely investigated and shown to export S1P in diverse cell types (Mitra *et al*., 2006; Gratschev *et al*., 2009). In mouse brain and spinal cord ECs, *abcc1*‐deficiency‐derived failure in S1P efflux terminates inside‐out S1P signalling to S1PR1, and abolishes the effects of S1P on basal P‐glycoprotein transport activity in the blood–brain and blood–spinal cord barriers (Cartwright *et al*., 2013). Moreover, exposure of human fibroblasts to dexamethasone increases SPHK1 expression, stimulates intracellular biosynthesis of S1P and upregulates ABCC1 to transport S1P to initiate signalling through S1PR3, and hence exerts a pronounced anti‐apoptotic action (Nieuwenhuis *et al*., 2009).

However, ABC transporters neither export S1P on their own in Chinese hamster ovary (CHO) cells nor participate in S1P export from erythroid cells (Hisano *et al*., 2011; Kobayashi *et al*., 2018), implicating cell‐type‐dependent S1P export activity.

#### (b) S1P transporter spinster homolog 2 (SPNS2)

SPNS2 is a major facilitator superfamily member and its role in exporting S1P was identified by *in vivo* study. In wild‐type zebrafish, at the yolk syncytial stage, myocardial precursors migrate towards one site to form a single heart. However, in *spns2* (*ko157*)‐mutant zebrafish, myocardial precursors showed a migration defect accompanied by a cardia bifida phenotype (Osborne *et al*., 2008; Kawahara *et al*., 2009). Given that the cardia bifida phenotype was also observed in S1PR2‐mutant animals (Kupperman *et al*., 2000), G_13_‐deficient embryos and embryos expressing dominant‐negative forms of Ras homologous guanine nucleotide exchange factor (RhoGEF, encoded by *arhgef11*) (Ye & Lin, 2013) and that interference with arhgef11 function suppresses the gastrulation defects caused by S1PR2 overexpression, it is proposed that S1P export from cells requires SPNS2 as a transporter and elicits signalling through S1PR2/G_13_/Rho during zebrafish myocardial migration (Fukui *et al*., 2014). A separate study showed that the cardia bifida phenotype was enhanced after overexpression of a lysophosphatidic acid (LPA)‐producing enzyme (autotaxin) in *spns2*‐deficient zebrafish, indicating that LPA signalling coordinates with S1P signalling during cardiac development (Nakanaga *et al*., 2014). Moreover, SPNS2 also functions together with the cell adhesion molecule fibronectin in regulating cardiac progenitor migration and lower jaw development (Hisano *et al*., 2013). In zebrafish, the SPNS2–S1PR2 axis is associated with proper growth and positioning in jaw skeleton development by inducing signalling molecule expression in facial ectoderm necessary for jaw development (Balczerski *et al*., 2012).

Consistent with the role of SPHK‐mediated S1P signalling in blood vessel formation, SPNS2 cooperates with S1PR1 and S1PR2 in zebrafish to regulate angiogenesis during proper embryonic vascular patterning (Mendelson *et al*., 2013). Moreover, SPNS2 was identified as a S1P transporter in mouse vascular ECs (but not in erythrocytes and platelets) (Hisano *et al*., 2012). Additionally, *Spns2*‐deficient mice showed hearing loss defects from 2 to 3 weeks after birth due to an early decline in endocochlear potential and progressive degeneration of hair cells in the organ of Corti (Chen *et al*., 2014). As another aspect of SPNS2‐dependent S1P signalling function, *Spns2*‐knockout mice are born with their eyes open, and they display abnormal postnatal retinal morphogenesis with disrupted cell polarity, delayed cell‐cycle exit of retinal progenitor cells and insufficient migration of newborn neurons, indicating an important role of S1P in postnatal retinal development (Bian *et al*., 2018). Mechanistically, S1P promotes eyelid closure by signalling through S1PR to activate ERK and epidermal growth factor receptor (EGFR)‐dependent mitogen‐activated protein kinase kinase kinase 1 (MEKK1)‐ Jun proto‐oncogene (c‐Jun) signalling and induce myocardin‐related transcription factor A (MAL) nuclear translocation, which in turn induces metalloprotease 10 (ADAM10)/ADAM17‐mediated EGFR signalling through upregulating expression of the microRNAs miR‐21 and miR‐22 (Bian *et al*., 2018). These data reveal diverse targets and mechanisms of S1P/SPNS2 during organ development in both zebrafish and mouse.

#### (c) Major facilitator superfamily transporters (MFSD2A and MFSD2B)

*Mfsd2a* and *Mfsd2b* encode the transmembrane proteins major facilitator superfamily (MFS) domain‐containing orphan transporters (MFSD2A and MFSD2B). Recent studies have shown that MFSD2A, but not MFSD2B, is required for the formation and maintenance of the blood–brain barrier in mouse brain, at least in part through its contribution to S1P export. MFSD2A does not transport S1P directly in brain ECs, however, MFSD2A binds S1P in the cell membrane and promotes S1P export by forming a protein complex with the major transporter SPNS2 (Wang *et al*., 2020), which in turn sustains an S1P‐rich microenvironment in the extracellular matrix and consequently, blood–brain barrier integrity. Deficiency in *Mfsd2a*, but not *Mfsd2b*, leads to breakdown of the blood–brain barrier in mouse embryos and adults, probably due to altered permeability and transcytosis in the brain ECs.

MFSD2B is mainly expressed in red blood cells and platelets, and shows S1P export activity in erythroid cells and CHO cells (Kobayashi *et al*., 2018). *Mfsd2b* knockout leads to a remarkable accumulation of S1P in red blood cells (RBCs) and reduction of plasma S1P in mice. This plasma S1P decrease does not affect blood vessel leakiness, but the mice become more sensitive to anaphylactic shock. *Mfsd2b*‐knockout mice exhibit anaemia with reduced RBC count and are more anaemic under stress‐induced erythropoiesis with RBCs showing membrane fragility and stomatocytosis. Thus, MFSD2B plays an important role in S1P export from red blood cells and platelets, and in maintaining the numbers and morphology of red blood cells (Vu *et al*., 2017).

Overall, S1P transporters are major components that finetune S1P levels and gradient across the cell membrane to enable inside–out signalling, and function to regulate the development of the heart, jaw, vascular system, eye and auditory system.

### (2) S1P receptors

**Fig 5:**
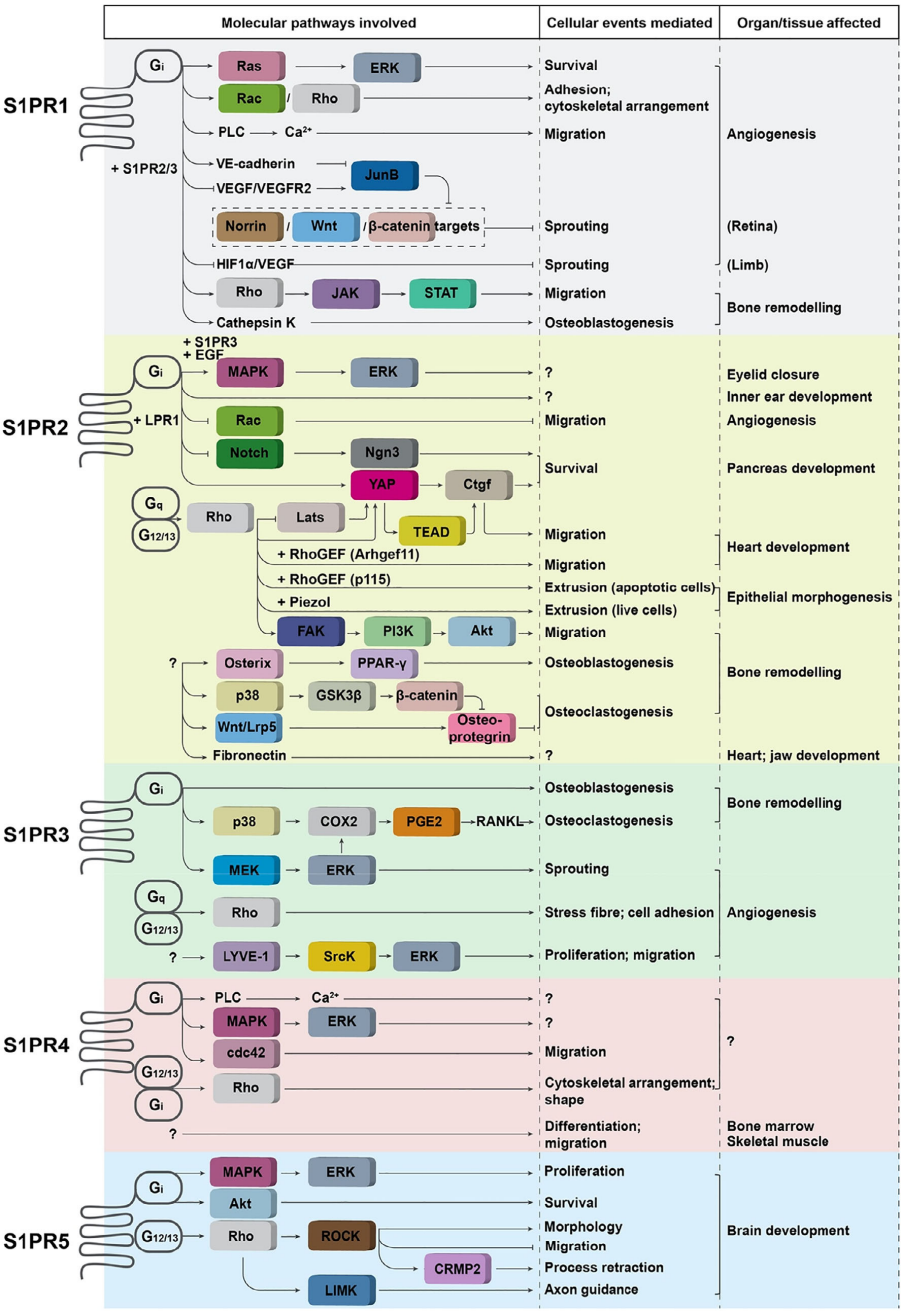
Sphingosine 1‐phosphate (S1P) receptor‐mediated extracellular mode of S1P signalling. S1P binds with five different G protein‐coupled receptors (S1PR1–5), each regulating a variety of downstream signalling pathways by coupling to different G proteins. These signalling pathways are directly linked to a series of cellular activities and are involved in the morphogenesis of various organs. Akt, protein kinase B; COX2, cyclooxygenase‐2; CRMP2, collapsin response‐mediated protein; EGF, epidermal growth factor; FAK, focal adhesion kinase; G_i_, G_q_ and G_12/13_, heterotrimeric G protein alpha subunits; GSK3β, glycogen synthase kinase 3β; HIF1α, hypoxia inducible factor 1 subunit alpha; JAK, Janus kinase; JunB, Jun B proto‐oncogene; LIMK, LIM kinase; LPR1, lipophorin receptor 1; Lrp5, lipoprotein receptor‐related protein 5; LYVE‐1, lymphatic vessel endothelial hyaluronan receptor 1; MAPK, mitogen‐activated protein kinase; MEK, mitogen‐activated protein kinase kinase; PGE2, prostaglandin E2; PI3K, phosphoinositide 3‐kinase; PLC, phospholipase C; PPARγ, peroxisome proliferator‐activated receptor gamma; Rac, rat sarcoma (Ras)‐related C3 botulinum toxin substrate; RANKL, receptor activator of NF‐kB ligand; Ras, rat sarcoma; Rho, Ras homolog family member; RhoGEF, Ras homologous guanine nucleotide exchange factor; ROCK, Rho‐associated coiled‐coil containing protein kinase; SrcK, Src proto‐oncogene kinase; STAT, signal transducer and activator of transcription; S1PR1–5, sphingosine 1‐phosphate receptors 1–5; TEAD, TEA domain; VE, vascular endothelia; VEGF, vascular endothelial growth factor; VEGFR2, vascular endothelial growth factor receptor 2; Wnt, wingless‐related integration site; YAP1, yes‐associated protein 1.

Five GPCRs (S1PR1–5) contribute to S1P signalling transduction (Spiegel & Milstien, 2003). S1PR1/2/3 receptors are widely expressed in various tissues, whereas the expression of S1PR4 is mainly confined to lymphoid and haematopoietic tissue and that of S1PR5 to the central nervous system, indicating they have overlapping as well as specific physiological functions (Sanchez & Hla, 2004). GPCR is bound to the α‐subunit of a heterotrimeric G‐protein (G_α_), shifting between inactive and active states through G_α_ binding with guanosine diphosphate (GDP) and guanosine triphosphate (GTP). G_α_ proteins are classified into four different isoforms: G_s_, G_i_, G_q_, and G_12/13_ (Okashah *et al*., 2019). S1P receptors have distinct G‐protein preferences, with S1PR1 coupling exclusively to G_i_, S1PR2 and S1PR3 to G_i_, G_q_ and G_12/13_, and S1PR4 and S1PR5 to G_i_ and G_12/13_ (Siehler & Manning, 2002). The functions of S1P receptors are summarized in Fig. 5, and their specific functions in angiogenesis and bone remodelling are depicted in Figs 3 and 4.

#### (a) S1PR1

Similar to SPHKs, S1PR1 functions in angiogenesis/vascular development through regulating cell adhesion, migration and survival in multiple *in vitro* angiogenesis models. In HUVECs, S1PR1 together with S1PR3 mediates S1P‐induced cytoskeletal reorganization and adherens junction assembly *via* Rho/Rac as well as S1P‐induced cell survival and morphogenesis through ERK signalling (Lee *et al*., 1999). S1P increases levels of N‐cadherin and VE‐cadherin, and decreases levels of angiopoietin 2 *via* S1PR1 in human retinal microvascular ECs (McGuire *et al*., 2011). S1P also prompts cell migration and capillary‐like tube formation in various angiogenesis models including HUVECs, human muscle vascular ECs (HMVECs) and bovine aortic ECs (Wang *et al*., 1999; Laurenzana *et al*., 2015), in part through S1PR1/G_i_/phospholipase C (PLC)/Ca^2+^ signalling pathways in human lymphatic ECs (Yoon *et al*., 2008). Matrigel plug assays in mice revealed that S1PR1/S1P stimulates outgrowth of new blood vessels (Chae *et al*., 2004b; Yoon *et al*., 2008; Laurenzana *et al*., 2015), highlighting a positive role of S1P in angiogenesis *in vivo*.

Angiogenic function of S1P/S1PR1 signalling was also demonstrated in mouse and zebrafish models. Both global and EC‐specific *S1pr1*‐knockout mice are embryonic lethal and die between E12.5 and E14.5, which initially was believed to be caused by incomplete vascular maturation with deficient coverage of vascular smooth muscle cells (VSMCs) and pericytes (Liu *et al*., 2000; Allende, Yamashita & Proia, 2003). Later detailed study suggested that the aortic VSMC phenotype occurs secondary to ectopic endothelial hyperplasia and vessel branching incompatible with embryonic survival. Furthermore, using the mouse retina model of angiogenesis together with S1PR1‐specific agonist and antagonists *in vivo*, *ex vivo* and *in vitro*, multiple studies revealed that S1PR1/S1P signalling functions to protect developing vasculature from aberrant angiogenic hypersprouting through stabilizing junctional vascular endothelial (VE)‐cadherin and inhibiting vascular endothelial growth factor A (VEGF‐A)/VEGF receptor 2 (VEGFR2) signalling (Gaengel *et al*., 2012; Jung *et al*., 2012). Moreover, S1PR1, S1PR2 and S1PR3 work redundantly during vascular development, with S1PR1 being the most important (Kono *et al*., 2004). *S1pr1/2/3* global triple‐knockout mice show severe retinal vascular defects resembling the hypersprouting phenotype in angiogenesis, with concurrent global transcriptome alteration and defective retinal vascular EC‐specific gene expression and differentiation, such as downregulation of a subset of Norrin/wingless‐related integration site (Wnt)/β‐catenin‐regulated genes. Detailed analysis further reveals that S1P coordinates with VEGF to induce Jun B proto‐oncogene (JunB) expression at the sprouting vascular front while acting with VE‐cadherin to suppress JunB expression in the nascent vascular network. This generates a spatial gradient of JunB, a component of the activator protein 1 family of transcription factors, which in turn enables retinal EC specialization (Fig. 3B) (Yanagida *et al*., 2020). During both angiogenesis and lymphangiogenesis, tip cells of ECs under laminar shear stress (LSS) transduced by the blood flow become quiescent and remodel into stable vessels. Note that during angiogenesis, in addition to blood‐derived S1P, S1PR1 can also be activated by LSS and regulates VE‐cadherin expression and the alignment of blood ECs in response to LSS (Jung *et al*., 2012). However, during lymphangiogenesis, S1PR1 is required neither for VE‐cadherin expression nor for tip cell alignment, but antagonizes LSS‐mediated VEGF‐C/VEGFR3 signalling to prevent the sprouting of lymphatic vessels *via* regulating claudin‐5 expression (Geng *et al*., 2020).

Notably, S1PR1/S1P also inhibits sprouting angiogenesis during limb vascular development either *via* the hypoxia/VEGF axis or in a VEGF‐independent manner (Chae *et al*., 2004a; Ben Shoham *et al*., 2012). Similarly, *S1pr1* knockdown leads to hypersprouting of the caudal vein plexus whereas *S1pr1* overexpression results in decreased sprouting in zebrafish (Ben Shoham *et al*., 2012; Mendelson *et al*., 2013). S1PR1 and VE‐cadherin also cooperate to inhibit VEGF‐driven angiogenic sprouting and promote vascular stabilization in zebrafish (Gaengel *et al*., 2012).

Similar to SPHKs, S1PRs participate in S1P‐mediated bone development. S1PR1 and S1PR2 coordinate to induce skeletal mesenchymal cell migration through activation of the RhoA/ Janus kinase (JAK)/signal transducer and activator of transcription (STAT) and RhoA/focal adhesion kinase (FAK)/PI3K/Akt signalling pathways, respectively (Quint *et al*., 2013). Moreover, S1P promotes osteoclast precursor monocyte migration, dynamically controlling bone mineral homeostasis in mice. Osteoclast/monocyte‐specific S1PR1‐knockout mice showed osteoporotic changes with increased osteoclast deposition on the bone surface due to S1P‐directed recirculation of osteoclast precursors from bone tissue to blood in response to altered chemotaxis (Fig. 4A) (Ishii *et al*., 2009). S1PR1 also participates in cathepsin K‐mediated bone formation in the mouse (Lotinun *et al*., 2013).

Additionally, the S1P/S1PR1/3 axis functions in zebrafish pancreas morphogenesis by directing insulin endocrine progenitors to migrate, aggregate and form the islet (Serafimidis *et al*., 2011). In the central nervous system, S1P promotes the proliferation of olfactory ensheathing cells *via* S1PR1/RhoA/Rho‐associated coiled‐coil containing protein kinase (ROCK)/yes‐associated protein (YAP) signalling and participates in the formation of the olfactory nerve layer, contributing to the development of the olfactory system (Bao *et al*., 2020).

#### (b) S1PR2

In contrast to S1PR1 and S1PR3 (Lee *et al*., 1999), in most cases S1PR2 negatively mediates EC migration and capillary tube formation partly by inactivating Rac (Laurenzana *et al*., 2015). Inhibition of platelet‐derived growth factor‐BB (PDGF‐BB)‐induced EC migration by the S1P/S1PR2/G_i_ axis depends on lipoprotein receptor‐related protein 1 (LRP1) and is mediated *via* inhibiting Rac1 (Nakajima *et al*., 2014). In zebrafish, as discussed above, S1PR2 functions together with SPNS2/S1PR1 in suppressing angiogenic sprouting and promotes vascular stabilization (Mendelson *et al*., 2013).

Interestingly, S1PR2 exerts a positive effect on myocardial migration during zebrafish heart tube formation, through activating RhoGEF/Arhgef11 and consequently, YAP1/TEA domain (TEAD)‐mediated connective tissue growth factor a (CTGFa), as well as interacting with fibronectin (Hisano *et al*., 2013; Ye & Lin, 2013; Fukui *et al*., 2014). Specifically, the large tumor suppressor kinase (LATS)/YAP1/CTGFa signalling pathway required for cardiac precursor cell migration has been delineated elegantly by several lines of evidence: (*i*) depletion of YAP1 and the YAP1/TEAD target CTGFa showed the same cardia bifida phenotype; (*ii*) endodermal overexpression of LATS1/2‐kinase‐resistant nuclear‐localized YAP1 rescued cardia bifida in *spns2* and *s1pr2* morphants; (*iii*) depletion of LATS1/2 rescued cardia bifida in *S1pr2* and G_13_ morphants; (*iv*) *yap1* mutants had a decreased expression of CTGFa and consequently enhanced endodermal cell apoptosis. Taken together, it has been proposed that SPNS2/S1PR2/G_13_ signalling regulates cardiac precursor cell migration during zebrafish heart development by inhibiting LATS1/2, resulting in nuclear translocation of YAP1 that functions together with TEAD to promote *ctgfa* expression (Fukui *et al*., 2014). However, how fibronectin fits into the SPNS2/S1PR2/G_13_/YAP1 pathway still awaits further investigation. Similarly, S1PR2 also coordinates with YAP/CTGF in regulating pancreas development. S1PR2 signalling functions in endocrine specification and lineage allocation through G_i_‐mediated YAP stabilization and Notch attenuation, in turn partially activating CTGF which participates in regulating the survival of endocrine and acinar progenitors (Serafimidis *et al*., 2017). The role of S1PR2 in pancreas morphogenesis is further confirmed by an endocrine clustering defect upon treatment with a mouse S1PR2 agonist as well as endocrine progenitor cell loss due to reduced cell survival upon treatment with a S1PR2 antagonist (Serafimidis *et al*., 2011).

S1P/S1PR2 signalling is also required for apoptotic and live epithelial extrusion. In normal conditions, the apoptotic cell produces and secretes S1P at the basolateral membrane that binds to S1PR2 on neighbouring cells, in turn, triggering Rho‐mediated intercellular actomyosin contraction through p115 RhoGEF and squeezing the cell out apically. Similarly, in response to crowding stress, the stretch‐activated channel Piezo1 triggers S1P‐Rho‐mediated live cell extrusion (Gudipaty & Rosenblatt, 2017). Therefore, epithelia with disrupted S1P synthesis or lacking S1PR2 cannot extrude cells apically and shift extrusion basally instead, as demonstrated by both *in vitro* cultured cell lines and *in vivo* zebrafish epidermis (Gu *et al*., 2011, 2015). S1P/S1PR2‐mediated cell extrusion provides a potential mechanism not only for tumour cell progression and invasion, but also for epithelial morphogenesis through dynamic cell rearrangement and cell fate specification (Ohsawa, Vaughen & Igaki, 2018).

Consistent with S1P signalling in bone remodelling, and in contrast to S1PR1 mediating osteoclast progenitors to migrate towards S1P (i.e. directing positive chemotaxis towards S1P), S1PR2 acts to direct chemorepulsion away from S1P (Fig. 4A), therefore S1PR2‐deficient mice exhibit decreased osteoclastic bone resorption and a reduced number of mature osteoclasts attached to the bone surface (Ishii *et al*., 2010). Moreover, despite the opposite effects of S1PR1 and S1PR2 on osteoclast migration, S1PR2‐mediated S1P signalling exerts a net positive effect on bone formation, i.e. stimulates osteoblastogenesis at the expense of adipogenesis by inversely regulating osterix and peroxisome proliferator‐activated receptor γ (PPARγ), and simultaneously inhibits osteoclastogenesis by inducing osteoprotegerin through the p38/glycogen synthase kinase 3β (GSK3β)/β‐catenin and WNT5A/LRP5 pathways (Fig. 4B) (Weske *et al*., 2018, 2019). Most interestingly, S1PR2‐mediated S1P signalling also inhibits differentiation of genuine adipose tissue stem cells, therefore *S1pr2* mice are osteopenic and obese (Weske *et al*., 2018, 2019).

*S1pr2* null mice are viable and fertile, whereas *S1pr2*;*S1pr3* double‐null mice display marked perinatal lethality (Ishii *et al*., 2002). Strikingly, both S1PR2 single‐ and surviving *S1pr2*;*S1pr3* double‐null mice show a profound hearing loss phenotype with progressive loss of vestibular function and degeneration of inner ear structure, probably due to failure in S1PR2‐induced blood supply to vasculature of stria vascularis in the inner ear (Herr *et al*., 2007; Kono *et al*., 2007), suggesting an important role of S1PR2 in the auditory system. In addition, S1PR2 shows redundancy with S1PR3 in murine eyelid development. Double, but not single, null mouse embryos show a defect in eyelid closure caused by a delay in epithelial sheet extension, likely due to attenuated EGF‐mediated mitogen‐activated protein kinase (MAPK)/ERK1/2 signalling (Herr *et al*., 2013).

#### (c) S1PR3

As discussed in Section III.2*a*, S1PR1 and S1PR3 function together to induce cell adhesion assembly and capillary‐like network formation during angiogenesis through G_i_/G_q_/G_13_‐coupled and Rho/Rac‐dependent signalling (Lee *et al*., 1999). Moreover, S1PR3 colocalizes and cooperates with lymphatic vessel endothelial hyaluronan receptor (LYVE‐1) to promote proliferation and migration of ECs, and thereby lymphangiogenesis through low molecular weight hyaluronan (LMW‐HA)‐induced tyrosine phosphorylation of Src proto‐oncogene (Src) kinase and ERK1/2 (Yu *et al*., 2015). Most interestingly, a synthetic 9‐amino acid peptide (KRX‐725), derived from the second intracellular loop of S1PR3 can mimic the effects of S1P to induce G_i_‐dependent ERK activation, and synergizes with protein angiogenic growth factor to promote sprout formation in angiogenesis through S1PR3‐mediated G_i_‐coupled mitogen‐activated protein kinase kinase (MEK)/ERK signalling in *in vitro* (human ECs and VSMCs), *ex vivo* (mouse aortic rings) and *in vivo* (mouse corneal pocket) studies (Licht *et al*., 2003).

*S1pr3*‐null mice are viable, fertile and superficially normal (Ishii *et al*., 2001), however, 8‐month‐old *S1pr3*‐deficient mice showed osteopenia and reduced bone formation (Keller *et al*., 2014). Moreover, *S1pr3* deficiency abolishes the osteoanabolic effects of S1P *in vitro* and also *in vivo* in the context of murine hormone calcitonin receptor (CTR) deficiency or administration of the nonselective S1P receptor agonist FTY720, demonstrating that S1PR3‐mediated S1P signalling acts downstream of hormone calcitonin (CT; inhibits S1P release from osteoclasts by suppressing SPNS2 expression) to regulate bone formation. S1PR3 exerts its function in part through initiating p38 and MEK1/ERK1/2‐mediated induction of the downstream osteoclastogenic cytokine RANKL (Fig. 4B) (Ryu *et al*., 2006; Keller *et al*., 2014). *S1pr2;S1pr3* double‐null mice cannot survive through infancy and have profound structural and functional defects in the auditory system and eyelid (Ishii *et al*., 2002; Herr *et al*., 2007, 2013; Kono *et al*., 2007).

#### (d) S1PR4

In CHO‐K1 cells, S1P induces cell migration through cell surface‐localized S1PR4 and G_i_‐mediated activation of CDC42 (Kohno *et al*., 2003). S1PR4 couples to both G_i_ and G_12/13_ and induces the Rho‐dependent pathway to regulate cytoskeleton rearrangement and cell shape in CHO‐K1 cells and to regulate cell migration in Jurkat T cells (Graler *et al*., 2003). The coupling of S1PR4 with G_i_ can also activate PLC (Graler *et al*., 2003) and the MAPK/ERK2 signal cascade (Van Brocklyn *et al*., 2000). *S1pr4* ^*−/−*^ mice are viable and fertile, but show an altered megakaryocyte morphology and defective platelet population in bone marrow. Together with *in vitro* observations of S1PR4 deficiency impeding proplatelet formation and S1PR4 overexpression promoting differentiation of human erythroleukemia cells to megakaryocytes, these data demonstrate a role of S1PR4 in terminal differentiation of megakaryocytes and repopulation of platelets (Golfier *et al*., 2010). S1PR4 together with S1PR1 is involved in S1P‐induced cell migration in skeletal muscle precursor cells (Cencetti *et al*., 2014).

#### (e) S1PR5

S1PR5 is expressed in oligodendrocytes of postnatal mouse brain and is localized to myelinated fibres and the myelin sheath. During brain development, S1P/S1PR5 induces cell proliferation through the G_i_‐dependent MAP/ERK pathway and cell morphological changes through the Rho kinase‐dependent ERK pathway in neural progenitor cells (cells that differentiate into neurons, astrocytes and oligodendrocytes) (Harada *et al*., 2004), and regulates migration of oligodendrocytes and oligodendrocyte precursor cells through the G_12/13_‐coupled Rho/ROCK signalling pathway (Novgorodov *et al*., 2007). S1P activation *via* S1PR5 induces process retraction of pre‐oligodendrocytes in immature cells through a Rho kinase/collapsin response‐mediated protein (CRMP2) signalling pathway and promotes the survival of mature oligodendrocytes through a pertussis toxin‐sensitive G_i_‐protein‐, Akt‐dependent pathway (Jaillard *et al*., 2005). In the *Xenopus laevis* visual system, S1PR5/S1P mediates retinal axon guidance through activation of RhoA and LIM kinase (Strochlic *et al*., 2008).

Through targeting five GPCRs, S1P exerts diverse functions during the development of multiple organs (Fig. 5). In many instances, these receptors exhibit consistent and overlapping functions. For example, S1PR1/2/3 cooperate to suppress hypersprouting angiogenesis during maturation of the vascular system (Fig. 3) and to promote bone formation (osteoblastogenesis) in bone remodelling (Fig. 4). On the other hand, they show distinct and sometimes even opposing functions under other circumstances. For instance, S1PR1 and S1PR3 promote whereas S1PR2 inhibits cell migration in angiogenesis (Fig. 3). Another example is in bone resorption processes where S1PR1 and S1PR2 mediate osteoclast circulation in opposite directions and S1PR2 and S1PR3 regulate osteoclastogenesis in opposing ways (Fig. 4). Most strikingly, S1PR2 exerts diverse and interesting functions, such as stimulating osteoblastogenesis and inhibiting osteoclastogenesis during bone remodelling. One setting in which S1PR2 generates seemingly paradoxical outcomes is in cell migration: it promotes cell migration during early cardiac development, but suppresses cell migration associated with vascular development. S1PR4 and S1PR5 elicit signalling involved in cell migration, cell shape and proliferation in distinct cell types.

## IV. ROLE OF INTRACELLULAR S1P SIGNALLING IN ORGAN MORPHOGENESIS

S1P is also known as an intracellular signalling molecule, regulating cellular activities involved in organ development such as cell proliferation and cell apoptosis by targeting TNF receptor‐associated factor 2 (TRAF2), heat shock protein 90α (HSP90α), heat shock protein 90β family member 1(GRP94), atypical protein kinase C (aPKC) and β‐site amyloid precursor protein (APP) cleaving enzyme‐1 (BACE1) and PPARγ in the cytoplasm, histone deacetylases (HDACs) and human telomerase reverse transcriptase (hTERT) in the nucleus, and prohibitin 2 (PHB2) in mitochondria (Fig. 2B).

### (1) Cytoplasmic targets

#### (a) TNF receptor‐associated factor 2 (TRAF2), HSP90α and GRP94

It has long been thought that TRAF2 interacts with SPHK1 to stimulate SPHK1 activity, which is required for TRAF2‐mediated activation of NF‐kB by TNFα signalling through S1P‐specific GPCR‐dependent extracellular S1P signalling (Xia *et al*., 2002). Surprisingly, SPHK1‐generated S1P was shown to function intracellularly and independently of its cell surface GPCRs for TNFα‐induced NF‐κB activation. S1P acts as a cofactor for the E3 ubiquitin ligase TRAF2, binds to TRAF2 at the N‐terminal RING domain and promotes TRAF2‐mediated polyubiquitination of receptor interacting protein 1 (RIP1) at lysine‐63, phosphorylation of inhibitor of nuclear factor kappa (IκB)‐kinase (IKKα/β) and IκBα, as well as IκBα degradation (Alvarez *et al*., 2010). Furthermore, upon ER stress the S1P–TRAF2–RIP1 complex can associate with the stress‐responsive proteins HSP90α, GRP94 and ER to nucleus signalling 1 (IRE1α), and likely also with the E3 ubiquitin ligase STIP1 homology and U‐box‐containing protein 1 (STUB1) *via* HSP90α, contributing to increased RIP1 polyubiquitination and activation of the NF‐κB pathway (Park *et al*., 2016). Similarly, S1P binds directly to the E3 ligase cellular inhibitor of apoptosis protein 2 (cIAP2) at the N‐terminal RING domain and promotes IL‐1‐induced polyubiquitination of interferon regulatory factor 1 (IRF1) at lysine‐63, which, in turn, activates a set of IRF1‐responsive genes (Harikumar *et al*., 2014).

#### (b) Atypical protein kinase C (aPKC)

aPKC is one of the three PKC subtypes, previously thought to be regulated only by protein–protein interaction (Steinberg, 2008). It is composed of a regulatory domain (C1), catalytic domains (C3, C4) for substrate binding and Pseudo and PB1 domains at its amino terminal. Recently, *in vitro* study found that in Hela cells intracellular, but not GPCR‐dependent extracellular, S1P activates aPKC by directly binding to its kinase domain, resulting in alleviation of autoinhibitory constraints. S1P‐mediated aPKC activation protects cells from apoptosis (Kajimoto *et al*., 2019).

#### (c) β‐site amyloid precursor protein cleaving enzyme‐1 (BACE1)

BACE1 is the enzyme for production of amyloid‐β peptide (Aβ/APP) in the nervous system, which is the main cause of Alzheimer's disease. APP is normally endoproteolysed by α‐secretase to sAPP‐α, with C83 later cleaved by γ‐secretase to generate P3 and APP intracellular domain (AICD). However, under disease conditions, APP is endoproteolysed by BACE1 (β‐secretase) to sAPP‐β and C99, and C99 then is cleaved by γ‐secretase to generate Aβ and AICD. Takasugi *et al*. (2011) demonstrated that S1P directly promotes BACE1 activity by binding to full‐length BACE1, therefore either down‐regulating SPHK1 or up‐regulating S1PL in mice decreases BACE1 activity and consequently reduces production of Aβ (Takasugi *et al*., 2011). *Sgpl1* deficiency‐induced S1P accumulation, in turn, impairs lysosomal degradation of APP and amyloidogenic C‐terminal fragments, and this deficit can be partially restored by selective mobilization of Ca^2+^ from ER or lysosomes (Karaca *et al*., 2014). Together, components of S1P signalling could serve as promising therapeutic targets for Alzheimer's disease.

#### (d) Peroxisome proliferator‐activated receptor gamma (PPARγ)

SPHK1‐generated S1P can interact directly with the transcription factor PPARγ through binding of the phosphate head group of S1P with the residue His322 in the ligand binding pocket of PPARγ. S1P induces expression of PPARγ and enhances the interaction and nuclear translocation of PPARγ and its putative coactivator peroxisome proliferative activated receptor 1β (PGC1β) in ECs. Both *in vitro* study and Matrigel assay reveal that S1P‐mediated PPARγ activity contribute to tube formation in angiogenesis and vascular development (Parham *et al*., 2015).

### (2) Nuclear targets

#### (a) Histone deacetylases (HDACs)

HDAC1 and HDAC2 are direct targets of S1P. Nuclear SPHK2 and SPHK2‐generated S1P directly binds with HDAC1 and HDAC2 in a co‐repressor complex at the promoter regions of cyclin‐dependent kinase inhibitor p21 or the transcriptional regulator c‐Fos, inhibits HDACs, enhances acetylation of histone H3 and subsequently enhances transcription (Hait *et al*., 2009). Reduced HDAC activity caused by nuclear S1P accumulation in *Sgpl1*‐deficient mouse embryonic fibroblasts is also linked with dysregulation of Ca^2+^ homeostasis (Ihlefeld *et al*., 2012).

Several lines of evidence have linked S1P‐mediated inhibition of HDAC with the development and function of the central nervous system. (*i*) *Sphk2* ^−/−^ mice, with reduced levels of S1P and dihydro‐S1P as well as histone acetylation in the hippocampus, exhibit a defect in memory function and contextual fear extinction, which can be rescued by the HDAC inhibitor suberoylanilide hydroxamic acid (Hait *et al*., 2014). (*ii*) Fingolimod (FTY720) can also be phosphorylated in mice, accumulates in the brain, binds and inhibits HDACs, enhances histone acetylation, regulates hippocampal memory‐related gene expression and facilitates fear extinction in severe combined immune‐deficient mice (Hait *et al*., 2014). Taken together, these studies suggest that a nuclear S1P–HDAC axis plays an important role in nervous system development and function probably through epigenetic regulation of gene expression.

#### (b) Human telomerase reverse transcriptase (hTERT)

SPHK2‐generated S1P physically interacts with hTERT binding of the C′ 3‐OH group of S1P and the Asp684 residue of hTERT and colocalizes with lamin B at the nuclear periphery. Nuclear S1P–TERT binding allosterically mimics phosphorylation of hTERT at Ser921 and prevents ubiquitination and proteasome degradation of hTERT mediated by the E3 ubiquitin ligase makorin ring finger protein 1 (MKRN1). Therefore, S1P binding to hTERT contributes to telomerase‐regulated biological functions, such as maintaining telomere integrity, promoting cell proliferation and delaying cellular senescence (Panneer Selvam *et al*., 2015).

### (3) Mitochondrial targets – prohibitin 2 (PHB2)

Strub *et al*. (2011) first identified that mitochondrial SPHK2‐generated S1P has a high binding affinity for PHB2, but not PHB1. SPHK2/S1P forms complexes and colocalizes with PHB2 in the inner membrane of mitochondria. The mitochondrial SPHK2/S1P–PHB2 interaction acts as a chaperone to regulate the assembly of electron transport chain complex subunit IV of cytochrome‐c oxidase and mitochondrial respiration in both Hela cells and cardiomyocytes from mouse heart (Strub *et al*., 2011). Furthermore, the mitochondrial SPHK2/S1P–PHB2 interaction contributes to cardioprotection by modulating oxidative phosphorylation and reactive oxygen species (ROS) production, thereby inhibiting permeability transition pore opening in preconditioning (Gomez *et al*., 2011). The mitochondrial SPHK2/S1P–PHB2 interaction is also implicated in protecting insulin‐secreting cells against cytokine toxicity, since a S1P decrease resulting from S1PL overexpression increases the expression of PHB2, which in turn increases ATP generation and oxygen consumption rate, therefore preventing the cytokine‐induced decrease in ATP levels and ER stress as well as apoptosis of insulin‐secreting β‐cells (Hahn *et al*., 2017).

Thus, emerging evidence indicates that S1P is a *bona fide* second messenger participating in cellular events involved in organ development. Given that SPHKs translocate between different cellular compartments, local S1P production may enable its binding with specific intracellular targets, thus exerting distinct cellular functions.

## V. ROLE OF S1P‐MEDIATED VESICULAR TRAFFICKING IN ORGAN MORPHOGENESIS

In addition to the signalling action of S1P either through GPCRs or intracellular targets, a more specific link of SPHKs and S1P with vesicular trafficking has emerged (Fig. 2C). In the *C. elegans* nervous system, SPHK1 is recruited to presynaptic membranes where locally generated S1P regulates both exocytosis (release of the neurotransmitter acetylcholine) and endocytosis (synaptic vesicle recycling) (Chan, Hu & Sieburth, 2012). SPHK1 enrichment at synaptic terminals is regulated by the muscarinic signalling pathway consisting of the muscarinic acetylcholine receptor GAR‐3, the heterotrimeric G protein G_q_, and its effector, TrioRhoGEF, in a Ca^2+^‐dependent manner involving calcium and integrin binding protein (Chan *et al*., 2012; Chan & Sieburth, 2012). Moreover, wild‐type human and *C. elegans* SPHK1, but not a membrane‐binding mutant of *C. elegans* (SPHK1V268Q), can rescue the neurotransmission defects of *sphk1*‐mutant worms, corroborating a conserved role of SPHK1‐dependent local conversion of sphingosine to S1P in vesicular trafficking (Shen *et al*., 2014). Consistently, SPHK1 is targeted to N‐terminal bin/amphiphysin/Rvs (N‐BAR) protein‐positive endocytic tubular invaginations upon perturbation of the cholesterol/sphingomyelin balance in the plasma membrane in COS‐7 and Hela cells. SPHK1 is also enriched in Ras‐related protein (Rab5)‐positive endocytic intermediates in Hela cells, at synapses in cultured hippocampal neurons and photoreceptor cells in mouse retina (Shen *et al*., 2014). Sphingosine or sphingosine‐like SPHK1 inhibitors induce a massive endosomal flux forming enlarged endosomes in multiple cell lines and mouse embryonic fibroblasts (Young *et al*., 2016; Lima, Milstien & Spiegel, 2017).

SPHK1/S1P‐mediated endocytic trafficking also converges with autophagy‐ and lysosome‐mediated degradation, as demonstrated by the observations that (*i*) decreased conversion of sphingosine to S1P causes disruption of autophagosome–lysosome fusion (Kajimoto *et al*., 2013) and defects in clearance of enlarged endosomes (Lima *et al*., 2017); (*ii*) the autophagic machinery coordinates with lysosome biogenesis to clear enlarged late endosomes in response to SPHK1 inhibition in mouse embryonic fibroblasts (Young *et al*., 2016); (*iii*) SPHK1 enhances autophagy biogenesis and function, and autophagy, in turn, stimulates SPHK1 relocalization to endosome/autophagosomes in neurons (Moruno Manchon *et al*., 2016). Furthermore, in *D. melanogaster* photoreceptors, deficiency in SPHK1 and overexpression of SPHK2 result in increased dihydrosphingosine 1 phosphate (DHS1P) levels relative to S1P, which in turn affects endolysosomal trafficking of the G protein‐coupled receptor rhodopsin and the transient receptor potential (TRP) channel and retinal degeneration in photoreceptors (Yonamine *et al*., 2011). Given the recognized role of endosomal/lysosomal trafficking in epithelial and neuron polarity, S1P‐mediated membrane dynamics and trafficking is expected to contribute directly to organ morphogenesis.

## VI. CONCLUSIONS

1. We are now beginning to understand the molecular mechanisms by which S1P exerts its function in organ morphogenesis. In contrast to the intracellular functions of S1P where S1P metabolic enzymes determine S1P activity directly, S1P extracellular activity is fine‐tuned through successive actions of transporters and receptors. Indeed, in many cases that have been investigated in detail recently such as cardiac precursor cell migration, angiogenesis and bone remodelling, orchestrated actions of almost every component along the S1P/SPHK/SPNS2/S1PR signalling axis and receptor subtype‐specific effects are required to ensure normal development and tissue homeostasis.
2. S1P activity is tightly controlled to maintain the balance between osteoclast‐mediated bone resorption and osteoblast‐mediated bone formation (Fig. 4). Firstly, at the level of abundance regulated by SPHK/S1PL, S1P activity suppresses osteoclastogenesis and stimulates osteoblastogenesis. Secondly, at the level of S1P extracellular or intracellular activity mediated by SPNS2, extracellular S1P potentiates osteoclast differentiation by acting on osteoblasts and intracellular S1P attenuates osteoclast differentiation. Thirdly, at the level of cell type‐dependent S1PR‐directed precise adjustments, while S1PR1/2/3 coordinate to promote osteoblastogenesis, S1PR1 and S1PR2 direct osteoclast progenitor migration in opposing directions, and S1PR2 and S1PR3 exert antagonistic effects on osteoclastogenesis.
3. Future studies must continue to define the comprehensive list of regulators by which cells modulate S1P abundance and compartmentalization. In contrast to the ‘bulk’ presence of structural components such as glycosphingolipids and sphingomyelins, S1P exists in extremely small quantities, therefore the cell requires a mechanism to sense and respond to subtle variations in S1P in the cytosol and subcellular pools. Some such growth factors, compounds, cytokines and proteins have been identified and described above, however, these probably only represent the tip of the iceberg. Obtaining the full spectrum of regulatory mechanisms will be important to understanding the system‐level regulation of S1P signalling.
4. It remains unclear how distinct transporters and receptors act in concert to transduce signals when multiple components are present simultaneously. It is unlikely that this occurs randomly, but rather is likely to take place under tight regulation. Characterization of the nature of these connections will be critical to understanding the properties and precision of finetuning S1P signalling.
5. Recent findings that HDAC1/2 are targets of S1P in the nucleus are exciting. Although further research is needed to consolidate these new findings, it is interesting to consider future prospects. Elucidating the molecular mechanisms underlying S1P‐mediated inhibition of HDAC1/2 would provide insights into the role of S1P signalling in cellular behaviours and organ development through epigenetic regulation of gene expression. Another interesting avenue to explore may be the possibility of using S1P and its mimetics as an HDAC1/2 inhibitor for the treatment of HDAC1/2‐associated diseases.
6. Despite the identification of multiple intracellular targets, our understanding of the intracellular targets of S1P remains rudimentary. In particular, what are the intracellular targets of S1P in organisms such as *D. melanogaster* and *C. elegans* where no extracellular mode for S1P is expected? Investigation of S1P signalling in these organisms may provide unique insights into S1P regulation and function.
7. A handful of S1P agonists and antagonists has been identified to interfere with S1P function in organ morphogenesis. Regardless of whether attention is targeted at the level of enzymes, lipid products or receptors, more S1P signalling‐based drugs could be explored and considered as promising therapeutic interventions for the treatment of diseases, especially given that S1P is a small molecule that can be administered directly.
